# Lingering effects of contraception management on feral mare (*Equus caballus*) fertility and social behavior

**DOI:** 10.1093/conphys/cox018

**Published:** 2017-03-18

**Authors:** Cassandra M V Nuñez, James S Adelman, Haley A Carr, Colleen M Alvarez, Daniel I Rubenstein

**Affiliations:** 1 Department of Natural Resource Ecology and Management, Iowa State University, Ames, IA, USA; 2 Ecology and Evolutionary Biology Department, Princeton University, Princeton, NJ, USA

**Keywords:** Contraception, feral horse, immunocontraception, management, prolonged subfertility

## Abstract

Due to the extirpation of their natural predators, feral horse populations have expanded across the United States, necessitating their management. Contraception of females (mares) with porcine zona pellucida (PZP) is a popular option; however, effects to physiology and behavior can be substantial. On Shackleford Banks, North Carolina, USA, treated mares have exhibited cycling during the non-breeding season and demonstrated decreased fidelity to the band stallion, but PZP's long-term effects on mare physiology and behavior remain largely unexplored. After the contraception program was suspended in this population, we examined how prior exposure to varying levels of PZP treatment impacted (1) foaling probability and foaling dates (a proxy for ovulatory cycling) from 2009 to 2014 and (2) mare fidelity to the band stallion and reproductive behavior during 2013 and 2015. Additionally, we evaluated the effects of time since the mares’ last treatment on these factors. Mares receiving any level of prior PZP treatment were less likely to foal than were untreated mares. Among mares that received 1–3 PZP applications, foaling probability increased with time since last treatment before declining, at ~6 years post-treatment. Mares that received 4+ applications did not exhibit a significant increase in foaling probability with time since last treatment. Moreover, previously treated mares continued to conceive later than did untreated mares. Finally, mares previously receiving 4+ treatments changed groups more often than did untreated mares, though reproductive behavior did not differ with contraception history. Our results suggest that although PZP-induced subfertility and its associated behavioral effects can persist after the cessation of treatment, these effects can be ameliorated for some factors with less intense treatment. Careful consideration to the frequency of PZP treatment is important to maintaining more naturally functioning populations; the ability to manage populations adaptively may be compromised if females are kept subfertile for extended periods of time.

## Teaser

Extended subfertility can cause changes in feral horse reproductive physiology and behavior. Here we examine long-term effects of contraception treatment on females even after cessation of treatment. We identify treatment schedules that have more/less pronounced effects and suggest management recommendations for future populations.

## Introduction

The extirpation of predator species in North America has precipitated the expansion of free-ranging ungulate populations (Eberhardt *et al*., 1982; McCullough *et al*., 1997), escalating human–wildlife conflict (Conover, 1995). Perhaps the most well-known example is that of increasing white-tailed deer (*Odocoileus virginianus*) populations across the United States. These deer damage crops, feast on suburban ornamentals, spread disease, and are involved in both vehicle and train collisions (Conover, 1997), resulting in human injury, the loss of human life and costs amounting to more than 1 billion USD of property damage per year (Mastro *et al*., 2008). Elk (*Cervus canadensis*) populations have also increased in some areas, with severe ecological impacts. In the Yellowstone ecosystem, unfettered elk populations have been associated with an ecological cascade of diverse effects including a reduction in available forage (Ripple and Beschta, 2003) and subsequent decreases in various vertebrate species including grizzly bears (*Ursus ortcos*) (Ripple *et al*., 2014) and various songbird species, including the Common Yellowthroat (*Geothlypis trichas*), Lincoln's sparrow (*Melospiza lincolnii*), and Warbling Vireo (*Vireo gilvus*) (Baril *et al*., 2011). Feral horse populations have also greatly expanded across the United Sates. In the west, feral horses and livestock compete for forage and water (BLM, 2011), while in the east, feral horses can negatively impact barrier island plant (Furbish, 1980) and estuarine (Levin *et al*., 2002) communities. Managers have few options with which to control these populations (e.g. culls, removals), and fewer still that are acceptable to the public (Hobbs *et al*., 2000; Porton, 2005). Consequently, contraceptive management has become increasingly popular (Hobbs *et al*., 2000; Porton, 2005).

A commonly used contraceptive in female ungulates, porcine zona pellucida (PZP), stimulates the production of antibodies that bind sperm receptors on the egg's surface, thereby blocking fertilization while still allowing ovulation and the associated cycles of sex hormones (Sacco, 1977). Because PZP does not affect hormone levels directly, it was previously suggested that PZP treatment did not significantly affect recipient behavior or physiology (Kirkpatrick *et al*., 1996, 1997; Powell and Monfort, 2001; Turner *et al*., 1997). However, research on white-tailed deer, elk and feral horses has shown that there can be unintended effects on reproductive behavior (McShea *et al*., 1997; Heilmann *et al*., 1998; Nuñez *et al*., 2009; Madosky *et al*., 2010; Ransom *et al*., 2010) and physiology (Nuñez *et al*., 2010; Ransom *et al*., 2013); in feral horses, these effects can be exacerbated in mares receiving higher numbers of total and/or consecutive PZP applications (Madosky *et al*., 2010; Nuñez *et al*., 2010). Briefly, studies investigating the response of free-ranging elk and white-tailed deer to PZP treatment indicate that recipient females extend reproductive behaviors into the post-breeding season (McShea *et al*., 1997; Heilmann *et al*., 1998). Authors suggest that in response to repeated unsuccessful mating attempts, females continue cycling in an attempt to gain additional reproductive opportunities (McShea *et al*., 1997; Heilmann *et al*., 1998). In feral horses, PZP-induced subfertility was also correlated with reproductive cycling in the non-breeding season (Nuñez *et al*., 2010; Ransom *et al*., 2013), increases in the amount of reproductive behavior directed towards mares by band stallions (Ransom *et al*., 2010), and an increase in group transfer and reproductive behavior by mares (Nuñez *et al*., 2009; Madosky *et al*., 2010). Few studies have documented how permanent such side-effects may be (Kirkpatrick, 1995; Madosky *et al*., 2010; Ransom *et al*., 2013) and to our knowledge, none have investigated whether the intensity of PZP management can influence female physiology and behavior after the suspension of treatment in managed populations.

Here, we examine the rate at which mares on Shackleford Banks, North Carolina, USA are able to return to their physiological and behavioral baselines after varying levels of contraception management and with increasing time since treatment. Specifically, we monitored per capita births and the effects of varying levels of PZP treatment on the likelihood of conception in individual mares, and foal birth dates from 2009 to 2014, during which time the contraception program was largely suspended. In addition, we examined mare group transfer and reproductive behavior in the summers of 2013 and 2015 to determine whether PZP-induced subfertility had any long-term behavioral effects on previously treated mares. This information is critical to understanding the full effects of long-term subfertility on individuals and populations, and will be integral to designing best-management practices for PZP contraception.

## Methods

### Study area

Shackleford Banks is a barrier island ~3 km off the coast of North Carolina, USA. The island measures ~15 km in length, and varies between 0.5 and 3 km in width. The Shackleford horses are of Spanish origin and were likely first introduced ~400 years ago (Conant *et al*., 2012). Currently, the population is co-managed by the National Park Service and the Foundation for Shackleford Horses (National Park Service, 2004). In 1997, federal law was instituted mandating the maintenance of at least 100–110 horses on Shackleford Banks (United States Congress, 1997). The National Park Service and the Foundation for Shackelford Horses limit the population to ~120 individuals, to reduce damage to the ecosystem.

### Study subjects

The reproductive units of Shackleford horses are typical of feral equids, consisting of coherent bands of one, or sometimes two or three stallion(s), with one to several mares and their offspring (Rubenstein, 1981). Double-male bands are present, though they make up a small portion of the Shackleford population (5–10% of bands during the course of this study). In addition, differences in the degree of band territoriality have been associated with the island's varying ecology, making the region in which animals reside a reliable proxy for social variation in this population (Rubenstein, 1981). Though the bands are predominantly non-territorial, individual bands are spatially distinct from one another (Feist and McCullough, 1976; Rubenstein, 1981, 1986). Interactions that occur between different bands typically involve younger individuals engaging in exploration and/or play, the dispersal of sub-adult individuals (both male and female), male–male assessments and/or contests, and the transfer of mares from one band to another (personal observation). Historically, band membership has been long lasting with most changes involving the dispersal of immature individuals. Band stallions sometimes fought to acquire mares from other groups, but stallions almost always retained their mares (Feist and McCullough, 1976; Rubenstein, 1981). More recently, PZP-induced subfertility has been associated with decreases in mare fidelity to the band stallion, increases in mare reproductive behavior, and mare cycling in what is typically the non-breeding season for these mares (Nuñez *et al*., 2009, 2010; Madosky *et al*., 2010).

### PZP contraception

The National Park Service began the application of PZP for the purposes of immunocontraception in January 2000. In 2009, the program was provisionally suspended because the population was deemed stable (Mason, 2017). The contraception of all but five previously treated mares was halted, and all previously untreated mares of reproductive age were given one dose and booster of PZP. All treatments were administered by the National Park Service from late February through April each year. Each injection contained 100 micrograms of PZP plus an adjuvant (mixed at the darting site). Initial doses contained Freund's Complete Adjuvant, Modified, *Mycobacterium butyricum* (Calbiochem #344 289). All subsequent boosters contained Freund's Incomplete Adjuvant (Sigma #F5506).

For the animals in this study, PZP deterred pregnancy in 99.91% of cases when administered during the same year (Stuska, 2014). These values are similar to those published for Assateague horses, in which PZP deterred pregnancy in 94% of cases (Turner *et al*., 1997). Over the course of the contraception program, ~19% of pregnancies were due to contraception failures. The contraception histories for the mares considered in this study are displayed in Table S1.

### Pregnancy and foaling

Fecal samples are collected by the National Park Service in January of each year. All pregnancy testing is completed by enzyme immunoassay of fecal material at the Science and Conservation Center at ZooMontana in Billings, Montana. Using the methods of Kirkpatrick *et al*. (1991), water extracts of fecal samples are assayed for estrone conjugates and nonspecific progesterone metabolites. In addition, the National Park Service has tracked the foaling records, including foal birth dates, for each mare since 2000 (Stuska, 2014). These data were used to supplement assay results.

### Behavioral and demographic sampling

Behavioral and demographic sampling was conducted primarily by two observers (C. Alvarez in 2013 and H. Carr in 2015) and was supplemented with data from three additional observers (B. Streater, 2013; M.A.F. Kent and K.E. Monroe, 2015). All observers were trained by C.M.V. Nuñez. We observed behavior in the majority of reproductive mares (aged 4 years and older) present during the study. We recorded group changing and reproductive behavior for 73% (43/59) and 95% (55/58) of mares in 2013 and 2015, respectively. The number of bands we observed comprised 68% (13/19) and 95% (18/19) of bands on the island in 2013 and 2015. Comparable to previous studies (Nuñez *et al*., 2009; Ransom *et al*., 2010), we observed mares for 7 weeks each year from mid-June to late August, totaling 350.15 hours of observation, averaging 3.13 h per mare (range = 1–6.05, SE ± 0.12). Horses were identified individually by color, sex, age, physical condition and other distinguishing markings including freeze brands. Ages are known from long-term records for the identified horses of Shackleford Banks (Nuñez, 2000).

We located each band an average of 0.87 times each week (range = 0.28–1.83, SE ± 0.03). We recorded its GPS location and composition, paying particular attention to the presence or absence of females. Group transfers were recorded if a mare was (a) not in the band she had been seen with previously, or (b) in a different band than she had been seen with previously. Mares did not need to remain with the ‘new’ group for a minimum length of time for the behavior to be considered a group transfer since even short-lived changes can affect band stability (Madosky *et al*., 2010) and mare stress physiology (Nunez *et al*., 2014). We remained with each group for a minimum of 30 min to ensure that individuals recorded as absent were not actually nearby, but out of our sight; when compared to 60-min field observations, 30 min ensured accurate data collection and allowed for larger sample sizes (Nuñez *et al*. 2010). We rarely witnessed mare transfer activity directly (*n* = 1); therefore, mare absence from a band was an important metric with which we measured the number of mare transfers between groups. On average, transfer behavior was confirmed by the mares’ presence in new bands within 12.32 days of mare absence (range = 2–31, SE ± 1.44).

All incidences of reproductive interest (including copulation, mounting, genital sniffing, and rump rubbing) directed to and initiated by mares were recorded *ad libitum* during scan sampling (Altmann, 1974) as in Nuñez *et al*. (2009).

### Statistical analyses

We analyzed data in R version 3.1.1. We used a linear mixed effects model (nlme package; Pinheiro and Bates, 2000) to determine whether the number of births had increased after the suspension of the contraception program (*n* = 157 total births; 112 births during the program; 45 births after program suspension). All births, regardless of mare age and treatment status at the time of foaling, were included in this analysis. Models included year as a random effect and the following fixed effects: mare treatment (previously treated/untreated), timing (during/after the contraception program), the interaction between mare treatment regime and timing, and a function allowing different variances across groups. We analyzed our data in both raw and arcsine-square root transformed formats as per Ahrens *et al*. (1990). The arcsine-square root transformation has been shown to correct deficiencies in the normality of error terms, homogeneity of variance, and additivity of percentage data (Ahrens *et al*., 1990).

To determine whether a mare's contraception history (number of total treatments received) affected her ability to foal in the years following contraception suspension, we used a generalized linear mixed model with a penalized quasi-likelihood (PQL) function (package MASS (Venables and Ripley, 2002)). The GLMM accounts for intra-subject correlation and individual heterogeneity (Jang and Lim, 2006) while the PQL function has proven reliable for the analysis of independent counts (Dean *et al*., 2004). Models included mare identity as a random effect and the following fixed effects: linear and second-order polynomial functions of mare age (to account for both linear and non-linear effects on the likelihood of foaling), mare contraception history (0 treatments/1–3 treatments/4+ treatments (Kirkpatrick and Turner, 2002), *n* = 68 mares, Table S1), and island region.

To test for differences in mares’ average foaling date with contraception history, we used linear mixed effects models, including a function allowing different variances across groups, and the following fixed effects: linear and second-order polynomial functions of mare age, mare contraception history (0 treatments/1–3 treatments/4+ treatments), and island region. We considered 37 births from 24 mares for this analysis (see Table S1).

To examine mare group changing and reproductive behavior, we analyzed the number of group changes mares made and the number of instances of reproductive interest both initiated and received by mares (*n* = 59 mares) over two field seasons conducted in 2013 and 2015 (see Table S1). We used linear mixed effects models including mare as a random factor, and the following fixed effects: linear and second-order polynomial functions of mare age, mare contraception history (0 treatments/1–3 treatments/4+ treatments), and island region. All behavioral data were Poisson distributed and log-transformed for analysis.

Parameters were removed from models by backwards elimination if *P* > 0.05. For each analysis, final models were run including linear and second- order polynomial functions of time since last treatment; for this analysis, only mares receiving at least one PZP treatment were considered (see Table S1). Mares less than 4 years of age and the 5 individuals that received treatment between 2009 and 2014 were excluded from all analyses save our per capita birth analysis (see above). In addition, where applicable, models were run both with and without mares belonging to double-male bands. Excluding these mares did not significantly change our findings; therefore, the models presented here include mares belonging to both single- and double-male bands. Where appropriate, we selected best fit models using Akaike's Information Criterion adjusted for small sample size (AICc) (Burnham and Anderson, 2002).

## Results

### Births

**Figure 1:**
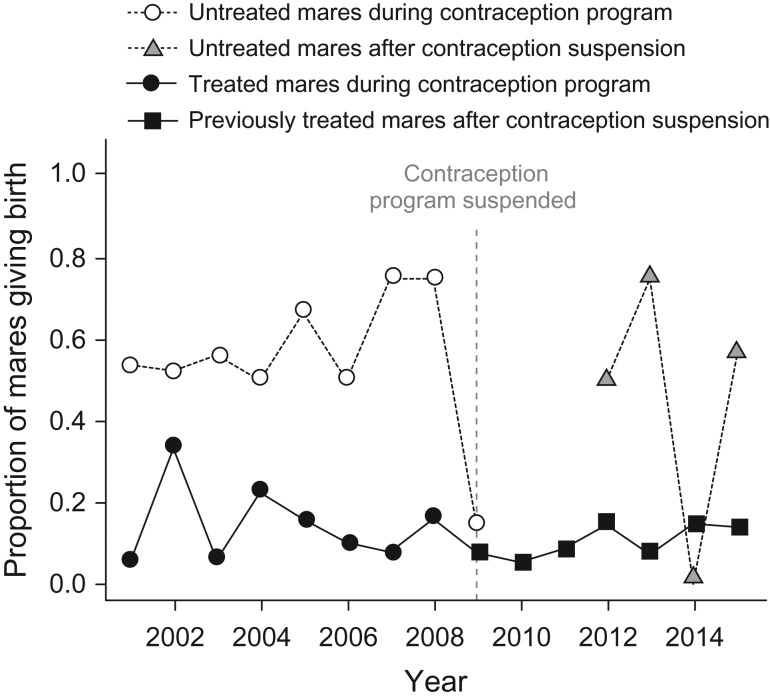
Proportion of mares giving birth in the years during and after contraception management. During the contraception program, treated mares (black circles) showed markedly lower birth rates than did untreated mares (open circles). In 2009, all untreated mares received PZP, leaving no untreated animals of reproductive age on the island in 2010 and 2011. Even after the suspension of treatment, previously treated mares (black squares) did not return to a level of fertility comparable to untreated mares before 2009 (open circles) or to younger, untreated animals that became reproductive between 2012 and 2015 (gray triangles).

Not surprisingly, yearly per capita births were considerably lower for treated mares than for untreated mares during the contraception program (Linear Mixed Effects Model: mare treatment regime (previously treated); estimate = −0.41, SE = 0.08, *t* = −5.30, *P* = 0.0003). This pattern did not change from 2009 to 2014 when the contraception program was largely suspended; previously contracepted mares continued to exhibit subfertility even after the cessation of treatment (Linear Mixed Effects Model: treatment group (previously treated) * timing (after contraception suspension); estimate = 0.07, SE = 0.13, *t* = 0.50, *P* = 0.62, Fig. 1). Results were similar with arcsine-square root transformed data (Ahrens *et al*., 1990) (Linear Mixed Effects Model: mare treatment regime (previously treated); estimate = −0.46, SE = 0.10, *t* = −4.61, *P* = 0.0007; treatment group (previously treated) * timing (after contraception suspension); estimate = 0.13, SE = 0.17, *t* = 0.74, *P* = 0.50). AICc analysis suggested that models without a variance function were a better fit to the data (AICc without variance function = 5.97 (raw), 24.19 (arcsine transformed); AICc with variance function = 8.89 (raw), 28.20 (arcsine transformed)).

### Contraception history and foaling after contraception suspension

**Figure 2:**
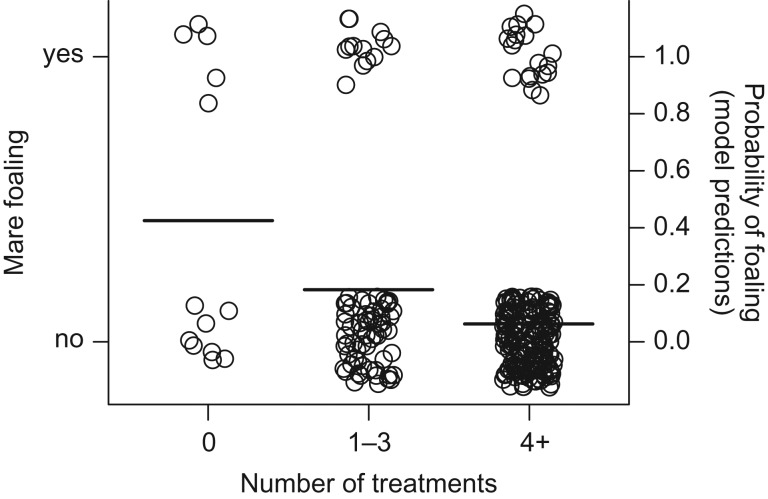
Probability of foaling and contraception treatment; circles represent individual births. Mares previously receiving any level of treatment were less likely to give birth than were untreated mares.

Even while controlling for the effects of mare age (Generalized Linear Mixed Model, mare age: estimate = 0.59, SE = 0.27, *t* = 2.20, *P* = 0.03; mare age^2^: estimate = −0.02, SE = 0.01, *t* = −2.18, *P* = 0.03), previously treated mares were less likely to give birth during the study period, regardless of the number of prior treatments received (Generalized Linear Mixed Model, number of treatments (1–3 treatments): estimate = −3.00, SE = 1.24, *t* = −2.40, *P* = 0.02; (4+ treatments): estimate = −4.19, SE = 1.27, *t* = −3.30 *P* = 0.001, Fig. 2). Island region had no effect on the probability of mare foaling (all *P* > 0.20). These results cannot be explained by inherent subfertility in mares slated for treatment; prior to contraceptive treatment, all mares exhibited equivalent fertility (Generalized Linear Mixed Model, treated vs. untreated: estimate = 0.36, SE = 0.43, *t* = 0.84, *P* = 0.41, Table S2).

**Figure 3:**
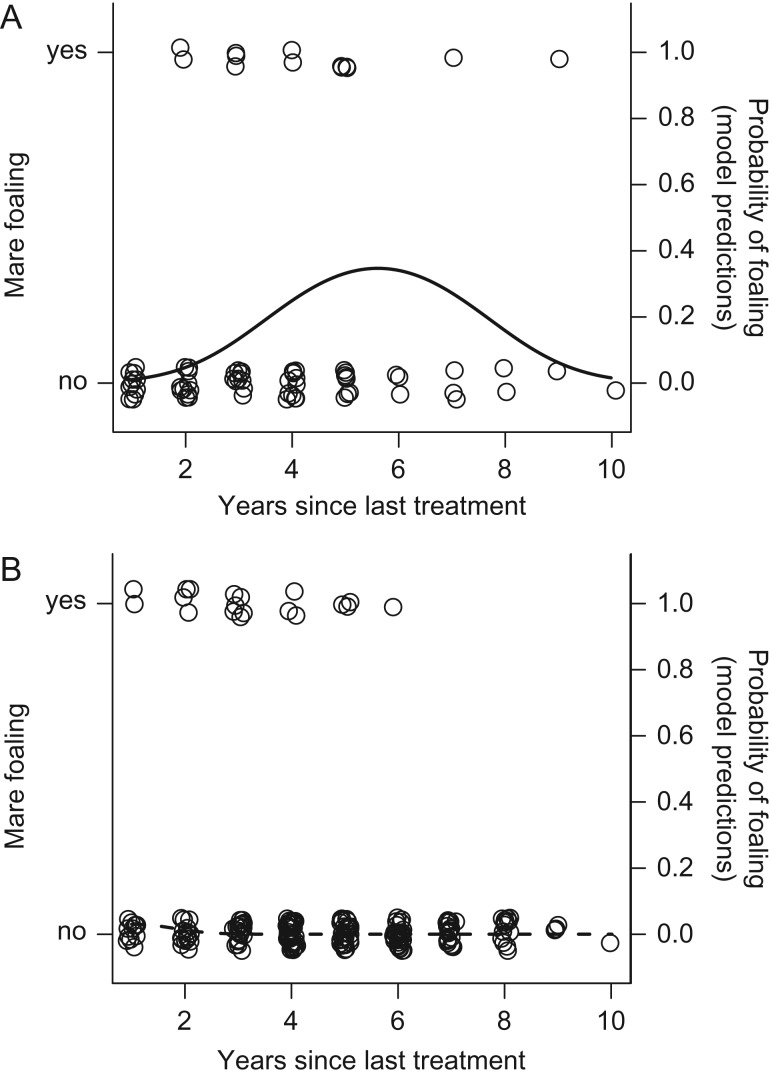
Probability of foaling after contraception suspension and mare treatment; circles represent individual births; solid and dashed lines indicate model predictions. Mares previously receiving 1–3 treatments (**A**) showed a gradual increase in foaling probability, peaking at ~6 years post-treatment, followed by a subsequent decrease. Mares previously receiving 4+ treatments (**B**) did not show a significant increase in foaling probability regardless of time since last treatment.

In addition, we sought to determine whether time since last treatment impacted the probability of foaling in previously treated mares. We found that mares previously receiving 1–3 treatments exhibited an initial increase in foaling probability, followed by a subsequent decrease (Generalized Linear Mixed Model, time since last treatment: estimate = 2.03, SE = 0.43, *t* = 4.67, *P* < 0.00001; time since last treatment^2^: estimate = −0.18, SE = 0.04, *t* = −4.07, *P* = 0.0001, Fig. 3A). Conversely, mares receiving 4+ treatments did not exhibit a discernable increase in foaling probability even after several post-treatment years (Generalized Linear Mixed Model, time since last treatment * number of treatments (4+treatments): estimate = −0.79, SE = 0.25, *t* = −3.14, *P* = = 0.002, Fig. 3B).

### Foaling date

**Figure 4:**
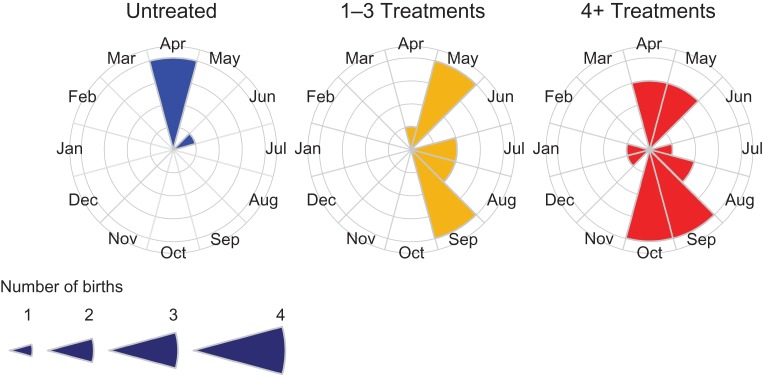
Average foaling date for untreated mares vs. previously treated mares. Mares previously receiving any level of treatment foaled later in the season than did untreated mares. Mares previously receiving 1–3 and 4+ treatments did not differ in their average foaling date.

Mares previously receiving any level of treatment gave birth later in the year than did untreated mares (Linear Mixed Effects Model, number of treatments (1–3 treatments): estimate = 2.29, SE = 0.78, *t* = 2.92, *P* < 0.00001; (4+ treatments): estimate = 3.50, SE = 0.90, *t* = 3.88, *P* < 0.0001, Fig. 4). Mares having received 1–3 treatments and 4+ treatments did not differ in their average foaling date (Tukey Honest Significant Difference Test: estimate = 1.21, SE = 0.87, *z* = 1.39, *P* = 0.34, Fig. 4). Island region and linear and second-order polynomial functions of both time since last treatment and mare age proved insignificant (all *P-*values > 0.08). Results suggested that models including a variance function were a better fit to the data (AICc with variance function = 155.56; AICc without variance function = 163.02).

### Mare fidelity and reproductive behavior

**Figure 5:**
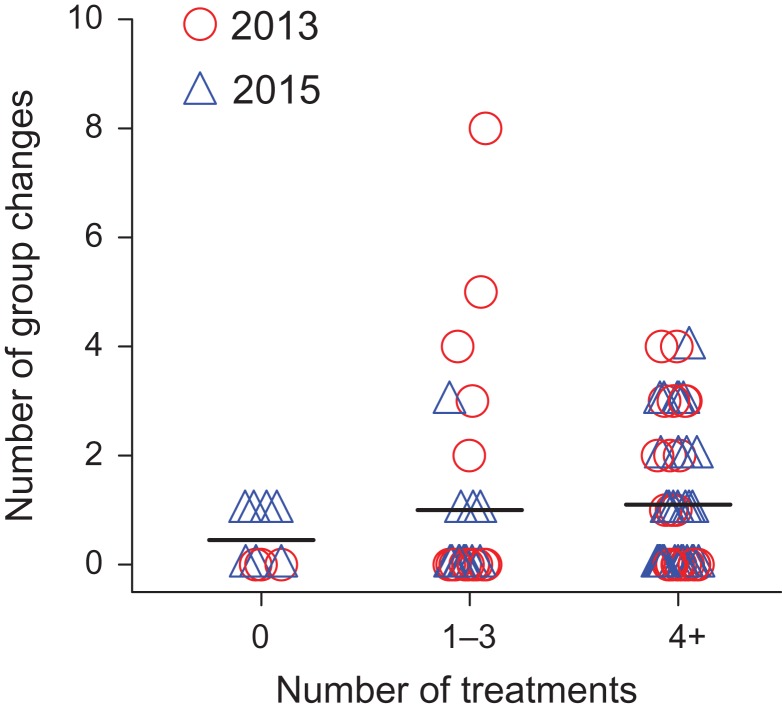
Number of group changes for untreated mares vs. previously treated mares in 2013 (red circles) and 2015 (blue triangles). Mares previously receiving 4+ contraceptive treatments made more group changes than did untreated mares. Mares previously receiving 1–3 treatments did not differ from untreated mares or those receiving 4+ treatments. Segmented lines indicate mean values.

Contraception history had a significant effect on mares’ group changing behavior. Even after controlling for the effects of mare age (Linear Model: estimate = −0.04, SE = 0.01, *t* = −3.04, *P* = 0.004), mares that previously received 4+ contraceptive treatments changed groups more often than did untreated mares (Linear Model: estimate = 0.75, SE = 0.27, *t* = 2.80, *P* = 0.007, Fig. 5). Mares that previously received 1–3 treatments were equivalent to both untreated mares (Linear Model: estimate = 0.43, SE = 0.26, *t* = 1.66, *P* = 0.10, Fig. 5) and those receiving 4+ treatments (Tukey Honest Significant Difference Test: estimate = 0.32, SE = 0.15, *z* = 2.04, *P* = 0.10, Fig. 5). Island region, linear and second-order polynomial functions of time since last treatment, and second-order polynomial functions of mare age proved nonsignificant (all *P-*values > 0.08). Results suggested that models without variance functions were better fits to the data (AICc without variance function = 206.55; AICc with variance function = 207.62).

Contraception history did not affect mare reproductive behavior (Linear Mixed Effects Model: all *P*-values > 0.10). Results suggested that models without variance functions were better fits to the data (AICc without variance function = 131.49; AICc with variance function = 133.76). Island region, linear and second-order polynomial functions of both time since last treatment and mare age proved insignificant (all *P-*values > 0.07).

## Discussion

We investigated the likelihood that previously treated mares on Shackleford Banks, North Carolina USA, would return to baseline fertility, foaling dates and behavior after the cessation of contraceptive treatment with PZP. Mares received varying numbers of total PZP inoculations, allowing us to more closely investigate the effects of treatment intensity on mare fertility and subsequent effects to mare behavior. This contraceptive has been administered to several mammalian species including, white-tailed deer (*O. virginianus*) (Miller *et al*., 1999), elk (*C. canadensis*) (Shideler *et al*., 2001), black bears (*Ursus americanus*) (Lane *et al*., 2007), bison (*Bison bison*) (Duncan *et al*., 2013), and African elephants (*Loxodonta africana*) (Fayrer-Hosken *et al*., 2000); these species have evolved an array of reproductive behaviors and social systems, which are vital to their survival and well-being. A more complete understanding of PZP contraception's potential long-term effects on recipient physiology and behavior is, therefore, of utmost importance if we are to manage and conserve species in the most effective and ethical manner possible. Feral horses have evolved a complex social structure, the maintenance of which is paramount to the overall health of group members (Kaseda *et al*., 1995; Linklater *et al*., 1999). Careful study of mare physiological and behavioral responses to long-term contraception management can serve as a model for other social species for which the formation and maintenance of social bonds is necessary for survival.

Even when controlling for the effects of mare age on fertility, previously treated mares were less likely to give birth than were untreated mares during the study period. Moreover, the number of treatments that mares previously received affected the likelihood that mares would return to fertility after treatment suspension. Regardless of age, mares that previously received 1–3 treatments were more likely to return to fertility than were mares that received 4+ treatments. These results cannot be explained by inherent subfertility in mares slated for treatment: prior to contraceptive treatment, all mares exhibited equivalent fertility. In addition, time since last treatment was an important predictor of foaling probability for mares that received 1–3 treatments, but not 4+ treatments. Mares receiving 4+ treatments remained largely subfertile even after several post-treatment years. Conversely, mares that received fewer treatments exhibited a gradual increase in foaling probability until ~6 years post-treatment, after which, foaling probability decreased. This decrease was likely due to the typical age-specific reproduction in feral mares (Garrott *et al*., 1991; Carnevale *et al*., 1994). Feral mares more than 15 years of age exhibit decreased foaling rates (Garrott *et al*., 1991) and pony mares at or older than 20 years of age are less likely to ovulate at regular intervals (Carnevale *et al*., 1994). In this study, mares that previously received 1–3 treatments were, on average, 12.42 years of age in 2009, when they received their last inoculation. At 6 years post-treatment, these mares would have been old enough to exhibit the reproductive senescence typical of the species. However, the fact that mares receiving fewer treatments were able to return to fertility while mares receiving more treatments were not is telling; these results demonstrate that mares can retain more natural physiological functioning with less intense contraceptive treatment.

Foaling date was also was also affected by contraception history. Mares previously receiving any level of treatment exhibited later foaling dates than did untreated mares. The persistence of this effect after treatment suspension may be related to increases in mare condition commonly associated with contraception treatment (Turner and Kirkpatrick, 2002). In domestic mares, increased body condition or ‘fatness’ has been associated with higher circulating levels of leptin (Fitzgerald and Bates, 2000). Mares exhibiting higher circulating leptin were more likely to exhibit reproductive activity during the winter months, suggesting that leptin may modify mare reproductive response to inhibitory photoperiod (Fitzgerald and McManus, 2000). Treated Shackleford mares exhibit the increased physical condition typically demonstrated by contracepted mares (Nuñez *et al*., 2010). It may be, therefore, that increases in body condition and concomitant increases in circulating leptin have contributed to prolonged effects on mare cycling even after contraception treatment suspension.

Potential effects of late birth dates to foal survival are difficult to assess on Shackleford Banks. In the past, animals perceived to be in poor condition have been removed from the island and adopted off, complicating the analysis of foal survival. As such, we cannot look to our data with confidence to determine if foal survival is compromised by later birth dates. Notably, foal survival is affected by birth date in western feral horses (Ransom *et al*., 2013). In these populations, the probability of foal survival decreases as parturition date moves further away from the summer peak in resources (Ransom *et al*., 2013), though these animals are undoubtedly subject to harsher environmental conditions than are Shackleford horses.

Contraception history also had an effect on mare fidelity to the band stallion. Mares that previously received 4+ treatments changed groups more frequently than did untreated mares. Mares that previously received 1–3 treatments did not differ from either untreated mares or those that previously received 4+ treatments. Combined with our findings on fertility, these results demonstrate that unintended side-effects of PZP treatment have the potential to become long term, i.e. they persist with continued subfertility, even if treatment, per se, has ended. However, our results also show that with less frequent treatment, some of these effects can be ameliorated with time, enabling more flexible population management.

Interestingly, our mare fidelity results are similar to those demonstrated in previous studies conducted during the contraception program (Nuñez *et al*., 2009; Madosky *et al*., 2010). In those studies, treated mares transferred groups more often than did untreated mares. These behaviors were linked to the presence of a foal (Nuñez *et al*., 2009): mares with foals, regardless of treatment status, tended to change groups less often, indicating that mares, having successfully reproduced with a stallion, were more likely to remain with said stallion and/or, that mares with foals required a more stable environment in which to raise offspring. In the present study, we found that mares that had previously received 4+ treatments changed groups more often than did untreated mares. The lower foaling probability exhibited by these mares, even after several post-treatment years, is likely the proximate mechanism behind this behavior, rather than PZP treatment, per se. These results show that changes in mare behavior formerly associated with active contraception treatment have persisted with prolonged subfertility, lending additional support to the assertion that such unintended side-effects can persist, even after active contraceptive treatment has ended. Moreover, such behavior represents a significant decrease in mare fidelity by previously treated mares. Prior to the contraception program, Rubenstein (1981) reported that across the entire island, only 10.8% of mares transferred groups, indicating high band fidelity by females. This is in stark contrast to what was found in our previous study, during which 53% of mares changed groups (Nuñez *et al*., 2009), in the 2 years of the Madosky study during which 66% and 73% of mares changed groups in 2007 and 2008, respectively (Madosky *et al*., 2010), and in our current study, during which 42% and 49% of mares changed groups, in 2013 and 2015, respectively.

Contrary to our results from an earlier study (Nuñez *et al*., 2009), previously treated mares did not engage in more reproductive behavior than did untreated mares. The disparity in our results may be due to differences in the timing of the two studies: the current study was conducted during the breeding season, when breeding behavior is relatively frequent amongst reproductive individuals; our previous study was conducted during the non-breeding season when breeding behavior is more variable. It is worth noting that in the Madosky study (2011), also conducted during the breeding season, Shackleford mares treated with PZP received more harassment from band stallions than did untreated mares. Taken together, these results could suggest that the amount of reproductive behavior in previously treated mares has returned to baseline levels. However, it is not clear whether ‘harassment’ (Madosky, 2011) is indicative of reproductive behaviors as we have defined them here. As such, our ability to compare results across time is limited.

Our results demonstrate the potential for long-term physiological and behavioral effects to PZP-treated individuals and that these effects can persist with continued subfertility, even after the cessation of treatment. However, our results also suggest that with less frequent treatment, these effects can be ameliorated with time, enabling more flexible population management.

Our results also contrast with claims that PZP is ‘completely reversible’; claims upon which feral horse management decisions have been defended (BLM, 2010). Similar long-term subfertility with intense PZP treatment has been found in feral horse populations living on Assateague Island National Seashore, MD (Kirkpatrick and Turner, 2002), in the closely related, but critically endangered Przewalski's horse (*E. ferus przewalskii*) (Feh, 2012), and in feral horses living in the western United States (CO and WY) (Ransom *et al*., 2013), indicating that these effects are not limited to specific sites or populations, but are pervasive amongst equids. In the Assateague study, nearly 70% of mares receiving three consecutive PZP treatments failed to demonstrate fertility for 1–4 years post-treatment (Kirkpatrick and Turner, 2002). In the Przewalski's horse, only 45% of 20 mares that had been treated with PZP for three consecutive years demonstrated fertility; those that did foal, did not do so until 6.7 years after their last treatment, on average. Moreover, four of nine females receiving only one treatment (one primer injection and one booster injection) did not produce offspring for 4+ years post-treatment (Feh, 2012). Though the mechanism behind the prolonged subfertility in Przewalski's mares is unknown, some of the Assateague mares demonstrated ovulatory failure (Kirkpatrick *et al*., 1992; Kirkpatrick and Turner, 2002). In western feral horse populations, previously treated mares were less likely to give birth and gave birth later into the calendar year for up to six post-treatment years, again demonstrating the potential for long-term side effects of prolonged, PZP-induced subfertility (Ransom *et al*., 2013).

The present study demonstrates that the intensity of treatment is also important: mares previously receiving 4+ treatments are less likely to return to fertility and exhibit decreased fidelity to the band stallion. Mares receiving any level of treatment are more likely to give birth later into the calendar year. Conversely, mares receiving 1–3 treatments are more likely to return to fertility and demonstrate behavior more typical of the species (Feist and McCullough, 1976; Rubenstein, 1981; Berger, 1986). These results largely support our previous assertion that more natural physiology and behavior can be maintained in feral horses with less aggressive contraception schedules (Nuñez *et al*., 2009).

Results similar to those demonstrated in this study have been documented in several captive species (Penfold *et al*., 2014). A retrospective analysis, combined with published literature and reliable anecdotal reports, demonstrated that extended periods of infertility in females can induce reproductive changes that negatively impact future fertility. This was the case for individuals treated with a contraceptive agent and also for those left untreated, but separated from male counterparts (Penfold *et al*., 2014). The mechanisms underlying these impacts are varied, but include endometrial hyperplasia [(Asian (*Elephas maximus*) and African elephants, Seba's bats (*Carollia perspicillata*), felids], uterine insufficiencies [wildebeest (*Connochaetes*)], asymmetric reproductive aging [white rhinoceros (*Ceratotherium simum*)], and enlarged ovaries and exuberant uterine responses [stingrays (*Myliobatoidei*)] (Penfold *et al*., 2014).

The close association with PZP-induced subfertility and mare behavior demonstrated here points to an important link between animal physiology and behavior that should be considered when implementing any management program. Alterations to an animal's reproductive physiology—for whatever managerial gain—will likely be associated with concomitant changes in behavior (McShea *et al*., 1997; Heilmann *et al*., 1998; Nuñez *et al*., 2009; Gilman *et al*., 2010; Madosky *et al*., 2010; Ransom *et al*., 2010). The effects of these subsequent changes—both to management program efficacy and to animal welfare—ought to be weighed against the perceived advantages of our physiological manipulations.

The broader implications of this research are notable. Because other methods for controlling feral horse populations can have pronounced side effects and are often unpopular with the public, PZP is undoubtedly an important option in a manager's toolkit (Hobbs *et al*., 2000; Kirkpatrick and Turner, 2003; Porton, 2005). However, our results suggest that the best use of this tool will balance attaining target population sizes against over-treating individual animals. Although the prolonged effects of PZP contraception outlined here could actually assist managers in keeping population sizes low (in feral horses and other over-abundant wildlife), the use of PZP with small populations and/or those of conservation concern should be approached with caution (Ransom *et al*., 2014). For instance, the fact that mares do not easily return to fertility after the cessation of treatment could limit the ability of populations to rebound in the face of stochastic declines. In the case of Shackleford Banks horses, prolonged PZP-induced subfertility has likely reduced the ability to adaptively manage the population; stopping treatment has not resulted in increased fertility, particularly for mares that previously received 4+ treatments. Given these results and the prolonged effects of PZP-induced subfertility on foaling schedules and behavior (and the potential for additional effects to non-recipient animals (Ransom *et al*., 2010)), we suggest that monitoring individual treatment histories, or modeling the statistical probability of multiple treatments per individual, could prove critical in maintaining more natural physiological and social dynamics, all while allowing for maximal flexibility in attaining management objectives.

## Supplementary material

Supplementary material is available at *Conservation Physiology* online.

## Supplementary Material

Supplementary Data
