# Supplementary material for: Lingering effects of contraception management on feral mare (Equus caballus) fertility and social behavior
Source: Conserv Physiol. 2017 Mar 18;5(1):cox018. doi: 10.1093/conphys/cox018 (PMC6007543; doi:10.1093/conphys/cox018)
Supplement: Supplementary Data [file cox018_suppl_1.zip › AltSupplementaryTable1_020161117.docx]

**Supplementary Material**

**Table S1**. Mare contraception history and analyses inclusion. “TSLT” indicates “time since last treatment”. Xs indicate mare inclusion in the corresponding analyses.

|  |  |  | **Statistical analyses** | | | | | | | |
| --- | --- | --- | --- | --- | --- | --- | --- | --- | --- | --- |
| **Mare ID** | **Mare age in 2009** | **Total**  **number of treatments** | **Foaling probability** | | **Foaling date** | | **Group changes** | | **Reproductive behavior** | |
|  |  |  | **no**  **TSLT** | **with TSLT** | **no**  **TSLT** | **with TSLT** | **no**  **TSLT** | **with TSLT** | **no**  **TSLT** | **with TSLT** |
| Alexa | 13 | 6 | X | X | X | X | X | X | X | X |
| Anastasia | 10 | 5 | X | X | X | X |  |  |  |  |
| Annie | 24 | 7 | X | X |  |  |  |  |  |  |
| Ariel | 11 | 4 | X | X |  |  | X | X | X | X |
| Biff | 17 | 1 | X | X |  |  | X | X | X | X |
| Bo | 13 | 2 | X | X |  |  | X | X | X | X |
| Bridget II | 11 | 5 | X | X |  |  | X | X | X | X |
| Carmen | 6 | 4 | X | X | X | X |  |  |  |  |
| Carrot | 17 | 1 | X | X | X | X | X | X | X | X |
| Chacha | 5 | 0 | X |  | X |  | X |  | X |  |
| Daisy | 15 | 8 | X | X | X | X | X | X | X | X |
| Darcy | 16 | 4 | X | X |  |  | X | X | X | X |
| Darkface | 19 | 7 | X | X |  |  | X | X | X | X |
| Delphi II | 7 | 4 | X | X |  |  | X | X | X | X |
| Dendera | 5 | 0 | X |  |  |  | X |  | X |  |
| Desdamona | 4 | 0 | X |  | X |  | X |  | X |  |
| Djibouti | 7 | 4 | X | X |  |  | X | X | X | X |
| Donoma | 3 | 2 | X | X |  |  | X | X | X | X |
| Doobie | 21 | 8 | X | X |  |  |  |  |  |  |
| Dotu | 10 | 5 | X | X |  |  | X | X | X | X |
| Dumey | 16 | 5 | X | X | X | X | X | X | X | X |
| Dusty | 14 | 6 | X | X |  |  | X | X | X | X |
| Hallie | 26 | 6 | X | X |  |  |  |  |  |  |
| Hardee | 12 | 5 | X | X | X | X | X | X | X | X |
| Havana | 4 | 0 |  |  |  |  | X |  | X |  |
| Hedvig | 5 | 1 | X | X | X | X | X | X | X | X |
| Helena | 15 | 6 | X | X |  |  | X | X | X | X |
| Hercules | 13 | 1 | X | X | X | X |  |  |  |  |
| Hermione | 4 | 3 | X | X | X | X | X | X | X | X |
| Hezekiah | 12 | 7 | X | X | X | X | X | X | X | X |
| Himalaya | 7 | 4 | X | X |  |  | X | X | X | X |
| Hiphop | 5 | 0 | X |  | X |  | X |  | X |  |
| Hoorah | 4 | 3 | X | X |  |  | X | X | X | X |
| Jaquincoke | 11 | 5 | X | X |  |  | X | X | X | X |
| Julie | 16 | 1 | X | X | X | X | X | X | X | X |
| Juniper | 19 | 4 | X | X |  |  | X | X | X | X |
| Keller | 2 | 1 | X | X | X | X | X | X | X | X |
| Kelty | 14 | 1 | X | X |  |  | X | X | X | X |
| Larissa | 8 | 4 | X | X |  |  | X | X | X | X |
| Lassie | 10 | 7 | X | X |  |  | X | X | X | X |
| Liani | 5 | 4 | X | X |  |  | X | X | X | X |
| MissIsabel | 6 | 3 | X | X |  |  | X | X | X | X |
| Noir | 15 | 2 | X | X |  |  | X | X | X | X |
| Paula | 13 | 4 | X | X |  |  | X | X | X | X |
| Penelope II | 9 | 4 | X | X |  |  | X | X | X | X |
| Persia | 7 | 4 | X | X |  |  | X | X | X | X |
| Sabrina | 9 | 7 | X | X | X | X |  |  |  |  |
| Sadie | 11 | 8 | X | X | X | X | X | X | X | X |
| Sarah | 26 | 7 | X | X |  |  | X | X | X | X |
| Sawathu | 9 | 2 | X | X |  |  | X | X | X | X |
| Selena | 4 | 0 |  |  |  |  | X |  | X |  |
| Serenac | 14 | 7 | X | X |  |  | X | X | X | X |
| Shag | 12 | 5 | X | X | X | X | X | X | X | X |
| Shiphrah | 6 | 4 | X | X |  |  | X | X | X | X |
| Shira | 8 | 4 | X | X |  |  | X | X | X | X |
| Slug | 19 | 7 | X | X |  |  | X | X | X | X |
| Sprite | 6 | 0 | X |  |  |  | X |  | X |  |
| Swing | 4 | 0 | X |  | X |  |  |  |  |  |
| Sydney | 14 | 2 | X | X | X | X | X | X | X | X |
| Tahiti | 7 | 5 | X | X | X | X | X | X | X | X |
| Tatya | 8 | 4 | X | X |  |  | X | X | X | X |
| Texas | 19 | 6 | X | X |  |  | X | X | X | X |
| Thunder | 8 | 4 | X | X |  |  | X | X | X | X |
| Tiger | 11 | 5 | X | X |  |  | X | X | X | X |
| Tuna | 27 | 9 | X | X |  |  |  |  |  |  |
| Wallace | 10 | 1 | X | X | X | X | X | X | X | X |
| Waltz | 4 | 0 | X |  | X |  |  |  |  |  |
| Wanaka | 7 | 4 | X | X |  |  | X | X | X | X |
| Wire | 16 | 4 | X | X |  |  | X | X | X | X |
| Zelda | 21 | 2 | X | X |  |  |  |  |  |  |
